# Peripheral Nerve Stimulation for Treatment of Headaches: An Evidence-Based Review

**DOI:** 10.3390/biomedicines9111588

**Published:** 2021-10-31

**Authors:** Steven Zhou, Nasir Hussain, Alaa Abd-Elsayed, Racha Boulos, Mohammed Hakim, Mayank Gupta, Tristan Weaver

**Affiliations:** 1 Department of Anesthesiology, Wexner Medical Center, The Ohio State University, Columbus, OH 43210, USA; steven.zhou@osumc.edu (S.Z.); racha.boulos@osumc.edu (R.B.); mohammed.mushtaqahmedhakim@osumc.edu (M.H.); 2 Department of Pain Medicine, Beth Israel Deaconess Medical Center, Harvard, Boston, MA 02215, USA; nhussai2@bidmc.harvard.edu; 3 Department of Anesthesiology, University of Wisconsin School of Medicine and Public Health, Madison, WI 53715, USA; abdelsayed@wisc.edu; 4 Department of Anesthesiology, Kansas City University of Medicine and Biosciences, Kansas City, MO 64106, USA; mayank.g@kansaspainmanagement.com

**Keywords:** peripheral nerve stimulation, headache, occipital nerve, vagus nerve

## Abstract

Headaches are one of the most common medical complaints worldwide, and treatment is often made difficult because of misclassification. Peripheral nerve stimulation has emerged as a novel treatment for the treatment of intractable headaches in recent years. While high-quality evidence does exist regarding its use, efficacy is generally limited to specific nerves and headache types. While much research remains to bring this technology to the mainstream, clinicians are increasingly able to provide safe yet efficacious pain control.

## 1. Introduction

Headaches are one of the most common medical complaints worldwide, with a prevalence rate of 46% in the general population [1]. A majority of headaches are of the migraine, tension, and cluster subtype, but many other forms exist. The healthcare burden of this is profound, as headaches consistently rank as the fourth or fifth most common reason for emergency-department visits in the United States, and they make it into the top 10 most disabling conditions according to the World Health Organization (WHO) [2]. There is also a significant economic impact from the burden of disease and the often-high cost of treatment. Unfortunately, diagnosis can be difficult, due in part to the complexity of its classification, contributions of referred pain from the spine, and presence of craniofacial pain syndromes. As our knowledge of the nervous system and pathophysiology of headaches and craniofacial pain has advanced, therapeutics have steadily moved towards directly targeting nerves of interest. After conservative measures have been exhausted, peripheral nerve stimulation (PNS) and field stimulation (PNFS) have emerged as a novel therapy for intractable headaches. Given the relatively recent advent of neuromodulation, this review seeks to help clinicians better understands the efficacy of PNS for headaches. We hope to summarize the high-yield evidence for the physician caring for these patients and provide an evidence-based overview of treatment strategies.

## 2. Classification

Headache disorders and craniofacial pain are often difficult to treat due to the confluence of the spine and both ascending/descending neural pathways in the region. Based on the International Classification of Headache Disorders (ICHD), headaches are generally classified as primary (migraine, tension-type, and cluster headaches) and secondary (caused by underlying systemic or neurologic conditions, trauma, medication overuse, substance withdrawal, and others) (Table 1) [3]. The location, duration, intensity, and characteristics of headache, along with associated symptoms, such as photophobia, phonophobia, nausea, and vomiting, are important in establishing a suitable diagnosis. Craniofacial pain includes lesions of specific nerves, such as the trigeminal, occipital, or vagus, which can lead to neuropathic pain. This overlap complicates the ability to tease out specific diagnoses from history alone, as presentations are often complex. Increasingly, physicians have utilized interventional techniques like PNS to treat all categories of headache disorders [4]. While some have shown promise, further data are needed to determine their true efficacy.

## 3. Conventional Management

Due to the complexity in etiology and presentation of headaches and craniofacial pain, treatment can be challenging. Typically, the first step in management is to explore and avoid known triggers, often in the form of caffeine, stress, poor sleep hygiene, and aspartame [5]. If pain continues despite this, the next step is typically pharmacological treatment in the form of oral medications, due to their relatively safe side-effect profile and simplicity of use.

Typically trialed in a stepwise fashion, escalations in management are made if no improvement in symptoms is seen. These are often measured by pain scoring (visual or otherwise) and by measures of disability, including days off work and days with symptoms. Oral pain medications are typically classified as either preventative (prior to an acute episode) or abortive (during an attack) [6]. While specific pharmaceuticals are used for the different headache subtypes, nonsteroidal anti-inflammatory drugs, anticonvulsants, selective serotonin reuptake inhibitors, tricyclic antidepressants, triptans, and corticosteroids are typically used as abortive medications [6,7]. Conversely, preventative therapies often include beta blockers, calcium channel blocks, and other anticonvulsants.

Despite multimodal approaches in management, many refractory cases resistant to conventional treatments subsist. Interestingly, medication overuse headaches have become increasingly prevalent, leading to a need for non-pharmacological interventions. Novel treatments pioneered and trialed in recent years include botulinum injections, nerve blocks, acupuncture, physical therapy, trigger-point injections, deep brain stimulation, and facet joint radiofrequency ablation for cervicogenic headaches [4,7]. While a thorough discussion of the evidence behind the techniques is outside the scope of this review, it is clear that there is no one-size-fits-all approach. Increasingly, PNS has been utilized as a minimally invasive intervention for these complex disorders. Evidence for its use is the primary focus of this review.

## 4. Peripheral Nerve Stimulation

### 4.1. Indications

While specific guidelines are sparse, the use of PNS has generally been indicated for chronic neuropathic pain disorders originating from specific peripheral nerves. The source of pain should logically follow that of a specific nerve distribution. While the exact mechanism of pain relief is unclear, it is thought to involve activation of central endogenous pain-modulation pathways [8]. As with any other pain modality, the success of PNS relies largely on patient selection. A typical patient for PNS is one who has failed conventional medical treatments and one for whom surgery has been deemed inappropriate [9]. After establishing a diagnosis and exhausting conservative treatment measures, patients are typically referred to a specialist for further discussion of symptoms, physician examination, and review of relevant imaging. Comorbid psychological illnesses should also be explored, as these can significantly reduce intervention success rates [10,11,12]. PNFS indications are even more nebulous, as these involve targeted stimulation of smaller mostly unnamed nerves at the site of pain [11]. Various targets and techniques are outlined below, in addition to the high-yield evidence for each.

### 4.2. Contraindications and Complications

There are generally few absolute contraindications for PNS. These typically include medical allergies, local infections, coagulopathies, immunocompromised status, comorbidities preventing fluoroscopic needle guidance, and patient refusal [11,13]. As these are peripheral procedures, the risk of permanent damage is relatively low. Complications are also similarly mild, but they can include headaches, muscle cramping, subcutaneous hematomas, seromas, and local skin infections [8]. Other common complications, along with their incidence seen in literature, are highlighted in Table 2 below. One of the most common themes in preventing success of treatment is improper lead placement or post-operative migration, causing displacement of electrodes from their target nerves [14]. While correctable, this is often frustrating for the patient and can worsen outcomes.

## 5. What Does the Evidence Suggest?

### 5.1. PNS Targets

**Figure 1:**
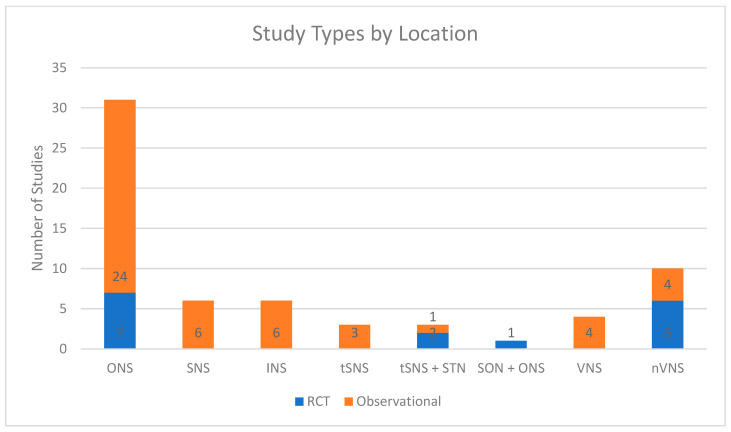
Breakdown of study types by location. ONS = occipital nerve stimulation, SNS = supraorbital nerve stimulation, INS = infraorbital nerve stimulation, tSNS = transcutaneous SNS, STN = supratrochlear nerve stimulation, VNS = vagus nerve stimulation, nVNS = noninvasive VNS.

**Figure 2:**
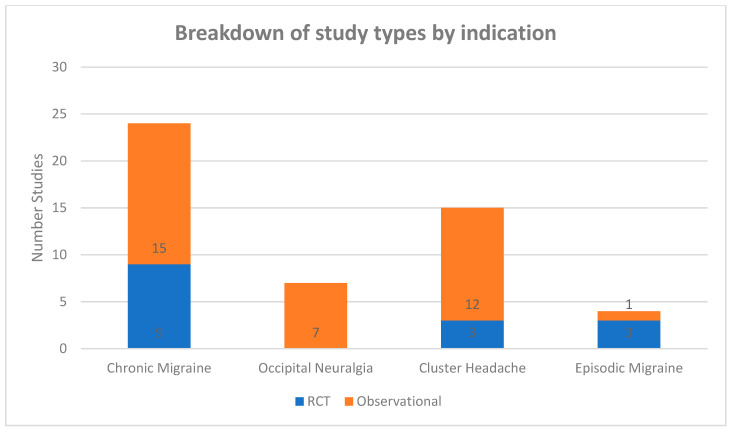
Breakdown of study types by indication.

There are numerous possible targets for PNS, with varying levels of evidence for each. Outlined is a high-yield summary of the common targets for headaches/craniofacial pain and broad evidence for their use (Table 3). These were further classified as high-quality evidence (primary from randomized controlled trials), moderate-quality (a mix of both randomized controlled trials and observational studies), or low-quality evidence (observational studies primarily from case reports or case series). For the physician making treatment decisions on these techniques, this quick guide can serve as a resource for decision-making on interventional approaches. Additional details regarding each high-quality trial are also presented for each stimulation technique. Overall, research tends to skew towards weaker observational studies versus higher-quality RCTs. Figure 1 breaks down the study types of location of stimulation, whereas Figure 2 breaks down the available literation by indication.

### 5.2. Occipital Nerve Stimulation (ONS)

One of the most well-studied targets for neurostimulation is the occipital nerve (Table 3). The occipital nerves are a group of nerves that originate from the C2/C3 spinal nerves and innervate a significant portion of the scalp, ear, and other nearby structures. The three major occipital nerves include the greater occipital nerve (GON), lesser (or small) occipital nerve (LON), and third (least) occipital nerve (TON) [75,76]. Common pathologies treated by occipital nerve stimulation include occipital neuralgia, chronic migraines, and cluster headaches (Table 4).

A review of the literature yielded a total of six randomized controlled trials, all of which involved the use of ONS for chronic migraines. Of these, three were industry-sponsored multicenter studies (PRISM study, ONSTIM, and Dodick et al.) [8,23,30]. The PRISM study was one of the first, but the results were published only as an abstract presentation with no formal manuscript submitted [23]. A total of 140 patients were randomized to either bilateral ONS or sham, with 125 completing the 12-week follow-up. No statistical difference was found between the treatment group and sham for reduction in migraines day per month. Later, Saper et al. completed ONSTIM, a multicenter feasibility trial where respondents were randomized to adjustable stimulation, preset stimulation, or medical management groups [30]. For the 66 patients who completed their headache diaries, 39% of patients receiving adjustable stimulation reported a >50% reduction in headache days or three-point greater decrease in average overall pain scores; symptom reduction on average was only 6% for preset stimulation, and 0% for medical management. Furthermore, Silberstein and Dodick et al. later completed a RCT with 157 patients at both the 12- and 52-week follow-ups [8]. While there was no significant reduction in VAS (i.e., by more than 50%) at 12 weeks, patients did report a significant reduction in headache days, headache reduction, and migraine-related disability. At 52 weeks, 59.5% of patients reported a 30% reduction in headache frequency or intensity, and 48% had an improvement of >50%. Overall, this study helped to cement the long-term viability of ONS for chronic migraines. In 2017, Mekhail et al. completed a single-center RCT, with 20 patients randomized to either an active or control group for 12 weeks, after which they received open-label treatment for an additional 40 weeks [17]. Overall, they found an overall average reduction in headache days per month by 8.51, with 60% of patients receiving >30% relief in headache days or pain intensity and 35% reporting >50%. Furthermore, MIDAS and Zung PAD scoring (migraine related disability) were reduced for all patients in the study.

Two single-site crossover RCTs have found similar results [32,33]. Serra and Marchioretto’s study randomized 30 patients with chronic migraines or medication-overuse headaches to either stimulation or sham, with a crossover after one month had passed or if headache had worsened [32]. Overall, they found significantly lower headache intensity and frequency in the stimulation arm, decreased MIDAS scores at the 1-year follow-up, significantly increased quality of life, and decreased triptan and NSAID use at each subsequent follow-up. Later, Slotty et al. evaluated various stimulation thresholds (effective stimulation, subthreshold, and no stimulation) in migraine patients already being treated with ONS and the importance of paresthesia [33]. They found a significant difference in pain (VAS) with suprathreshold stimulation (where patients reported paresthesia during stimulation) and subthreshold stimulation (1.98 ± 1.56 vs. 5.65 ± 2.11). Similar results were seen when comparing subthreshold stimulation and no stimulation (5.65 ± 2.11 vs. 8.45 ± 0.99). Overall, this study found that, while paresthesia was not required to achieve pain reduction, suprathreshold stimulation yielded better pain control, highlighting the importance of stimulator customization.

In 2017, Liu et al. published an RCT evaluating transcutaneous occipital nerve stimulation to prevent migraines [24]. This study randomized 110 patients into five groups who received transcutaneous ONS (tONS) at various frequencies (2, 100, and 2/100 Hz), underwent sham tONS, or received oral topiramate. Overall, they found that tONS at all frequencies, and topiramate, all yielded a significant reduction in headache days compared to baseline at the 3-month follow-up. However, only the tONS at 100 Hz and topiramate groups showed a significant difference when compared with sham. Interestingly, tONS, topiramate, and sham all led to decreases in VAS, potentially speaking to the placebo-effect of tONS. Overall, this study suggests that tONS may also be effective in the prevention of chronic migraines.

### 5.3. Trigeminal Nerve Stimulation

The trigeminal nerve carries sensory components for much of the head and innervates muscles in the lower jaw. It then divides into the ophthalmic (V1), maxillary (V2), and mandibular (V3) branches [78]. The ophthalmic further divides into the supraorbital nerve and the supratrochlear nerve, while the auriculotemporal nerve is a branch of the mandibular nerve.

Jiang et al. completed a RCT evaluating the combination of flunarizine and transcutaneous supraorbital neurostimulation (tSNS) on episodic migraines on patients randomized to flunarizine only, tSNS only, or both flunarizine and tSNS [55]. They found that while monthly migraine days was decreased in all three groups, pain reduction by >50% was greatest (78%) in the combination therapy group than either flunarizine (46%) or tSNS (39%) alone, showing the efficacy of pharmacological and interventional therapies in treating migraines (Table 5).

In 2019, Chou et al. conducted a multicenter RCT with 109 patients evaluating the efficacy of tSNS for acute migraine attacks. They found that one hour of tSNS treatment resulted in a 59% decrease in VAS versus a 30% decrease with sham [54]. Overall, these studies help support the efficacy of external neuromodulation as viable treatment for migraine headaches.

Several studies have also been conducted on the efficacy of neuromodulation on multiple concurrent nerve targets (Table 6). The first RCT to show the effectiveness of non-invasive supraorbital and supratrochlear PNS for migraines was completed by Schoenen et al. in 2013 [57]. In this study, 67 patients with at least two migraine attacks per month were randomized to either sham or stimulation with daily sessions of tSNS with Cefaly device. After 3 months of treatment, the stimulation group experienced a significant reduction in the average number of migraine days, with 38% achieving a >50% response. This study overall demonstrated a 26% therapeutic gain, which is within the range of those reported for other commonly used migraine treatments.

**Table 5:** Summary of studies reviewed regarding trigeminal nerve stimulation.

Author	Nerve	Study	Design	Results
Simopoulos et al. (2010) [79]	Auriculotemporal	Chronic Migraine n = 1	Observational	Case report, pain score decrease from 8–9/10 to 5/10 at 16 months, improved MIDAS
Vaisman et al. (2012) [53]	Supraorbital/supratrochlear	Trigeminal Autonomic Cephalgia n = 5	Observational	Decrease in average VAS of 1.6. 100% reported improvement in functional status for ADLs. 60% weaned off opioids
Johnson and Burchiel (2004) [49]	Supraorbital or infraorbital	Trigeminal Neuropathic Pain n = 10	Observational	70% of patients with >50% pain relief and medication use decline
Slavin et al. (2006) [51]	Supraorbital or infraorbital	Craniofacial Pain n = 7	Observational	68% with complete pain relief, although some patients received concurrent ONS
Amin et al. (2008) [48]	Supraorbital	Supraorbital Neuralgia n = 10	Observational	Overall, decreased headache scores, 50% decrease in opioid consumption up to 30 weeks
Stidd et al. (2012) [52]	Supraorbital or infraorbital (or both)	Trigeminal Neuropathic Pain n = 3	Observational	Postsurgical and posttraumatic patients with 100% resolution of pain, postherpetic neuralgia with 60%
Narouze and Kapural (2007) [50]	Supraorbital	Cluster Headache n = 1	Observational	Complete ission 14 months after implantation
Russo et al. (2015) [56]	Transcutaneous supraorbital	Migraine n = 24	Observational	75% of patients with >50% reduction of monthly migraine attacks and migraines days. Significant reduction in pain intensity and HIT-6
Jiang et al. (2018) [55]	Transcutaneous supraorbital	Episodic Migraine n = 154	Single center RCT	39% of patients with >50% reduction in migraine days with tSNS. 78% with >50% reduction with flunarizine with tSNS
Chou et al. ACME (2019) [54]	Transcutaneous supraorbital	Acute Migraine n = 106	Multicenter double-blinded RCT	59% decrease in acute migraine VAS for transcutaneous trigeminal nerve stimulation vs. 30% for sham

tSNS = transcutaneous supraorbital neurostimulation.

### 5.4. Vagal Nerve Stimulation

The vagus nerve plays a major role in the autonomic nervous system, regulating metabolic homeostasis, control of various organs/glands/muscles in the body, and mediating the transfer of sensory information throughout the body [80]. Historically, neuromodulation of this nerve has played a role in treating epilepsy and depression. In more recent years, there has been a movement towards both invasive and noninvasive methods of vagus nerve stimulation for the treatment of migraine and cluster headaches (Table 7).

**Table 6:** Summary of studies reviewed regarding combined PNS.

Author	Nerve	Study	Design	Results
Reed et al. (2010) [59]	Occipital and supraorbital	Chronic migraine n = 7	Observational	Full therapeutic response at 1–35 month follow-up
Deshpande and Wininger (2011) [81]	Occipital and auriculotemporal	Complicated migraine and occipital neuralgia n = 1	Observational	>50% reduction in headache onset at 24 month follow-up
Mammis et al. (2011) [82]	Occipital, supraorbital, infraorbital	Cluster headache n = 1	Observational	Decrease from 3–4 episodes per day to 3–4 per month at 36 month follow-up
Hann and Sharan (2013) [58]	Occipital and supraorbital	Chronic migraine n = 14	Observational	71% of patients with >50% reduction in pain severity
Schoenen et al. (2013) [57]	Transcutaneous supraorbital and supratrochlear	Chronic Migraine n = 67	Multicenter double-blinded RCT	Decrease in mean migraine days, >50% relief greater in intervention arm, reduced monthly attacks, monthly acute antimigraine medication use
Reed et al. (2015) [60]	Occipital and supraorbital	Hemiplegic migraine n = 4	Observational	Average headache frequency decreased by 92%, VAS by 44%, MIDAS by 98%, medication use by 96%

Gaul et al. conducted a large multicenter PREVA RCT in the UK, evaluating the use of noninvasive VNS as an adjunct to standard of care (oxygen and triptans) for chronic cluster headaches [69]. As one of the largest RCTs with a total of 97 patients, this study found a significantly greater reduction in the mean number of attacks per month (5.9 vs. 2.1) compared to standard of care. In addition, 40% of patients with the adjunct nVNS reported a greater than 50% reduction in pain as compared to just 8.3% for the control. Overall, this study suggests that nVNS has a beneficial role as an adjunct to standard of care for cluster headaches.

Silberstein et al. conducted the multicenter double-blinded RCT called ACT1 to evaluate the use of nVNS on acute treatment of episodic and chronic cluster headaches [73]. Interestingly, the study found a significantly higher response rate (pain relief based on scoring) for episodic cluster headaches versus the sham, but not for the chronic cohort. Goadsby et al. conducted a similar study on the European population and found comparable results [70]. This suggest that nVNS is more effective at treating episodic cluster headache than chronic cluster headache.

Subsequently, Silberstein completed the EVENT RCT to assess the use of nVNS for chronic-migraine prevention [67]. Patients were randomized to nVNS or sham, with treatments administered personally at a specific time of the day. Overall, they found no statistically significant difference at the 2-month follow-up. Following this, a small subset of patients completed a further 6 months of open-label treatment, where statistical significance (decreased headache days) was achieved. While limited, this suggests that continued nVNS use may have a benefit for chronic-migraine prevention.

Finally, Tassorelli et al. completed the PRESTO RCT to evaluate nVNS as an acute treatment for migraines [74]. Overall, this found that nVNS was superior to the sham in aborting the first treated attack of acute migraine at 30 and 60 min, with repeat-measures testing supporting superiority in pain freedom from 30 to 120 min. The pain-free response rate at 120 min was also similar to those achieved by oral triptans and NSAIDs, suggesting their usefulness for acute migraines.

**Table 7:** Summary of studies reviewed regarding vagus nerve stimulation.

Author	Nerve	Study	Design	Results
Hord et al. (2003) [61]	Invasive Vagus	Chronic migraine n = 4	Observational	All patients reported reductions in headache frequency and pain score
Mauskop (2005) [63]	Invasive Vagus	Chronic migraine, cluster headache n = 6	Observational	Significant reduction in cluster headaches in 2 patients, 2/4 migraine patients
Lenaerts et al. (2008) [62]	Invasive Vagus	Chronic migraine n = 10	Observational	80% of patients had >50% reduction in headache frequency, 50% completely headache free
Cecchini et al. (2009) [64]	Invasive Vagus	Chronic headache associated with depression n = 4	Observational	2/4 patients with improved headache and depression
Nesbitt et al. (2015) [72]	Noninvasive Vagus	Cluster Headache n = 19	Observational	79% of patients with improved headache intensity, 47% of attacks aborted after average of 11 min
Gaul et al. PREVA (2016) [69]	Noninvasive Vagus	Cluster Headache n = 97	Multicenter open-label RCT	Adjunct noninvasive VNS lead to significant reduction in attacks vs. standard of care, 40% patients >50% response vs. 8.3% for standard of care
Marin et al. (2018) [71]	Noninvasive Vagus	Cluster Headache n = 30	Observational	Mean attack frequency decreased from 26.6 per week to 9.5 per week after nVNS. Significant decrease in attack frequency, severity, duration
Goadsby et al. (2014) [83]	Noninvasive Vagus	Acute Migraine n = 27	Observational	Pain free rate at 2 h 21% for first attack, treated at 15 min intervals with nVNS
Barbanti et al. (2015) [65]	Noninvasive Vagus	Acute Episodic and Chronic Migraine n = 50	Observational	56% of patients with >50% reduction in VAS at 1 h, 64.6% at 2 h. 33% were pain free at 2 h
Straube et al. (2015) [68]	Auricular Transcutaneous Vagus	Chronic Migraine n = 40	Single center double-blinded RCT	Patients in 1 Hz group with significantly larger reduction in headache days than 25 Hz. 29% with >50% response for 1Hz
Kinfe et al. (2015) [66]	Noninvasive Vagus	N = 20 (10 for episodic migraine, 10 for chronic migraine	Observational	Significant reduction in VAS, mean headache days per month, and mean migraine attacks
Silberstein et al. ACT 1 (2015) [73]	Noninvasive Vagus	Cluster Headache n = 133	Multicenter double-blinded RCT	Significant response in pain score for those with episodic cluster headache vs sham. However, no total population difference found
Goadsby et al. ACT 2 (2018) [70]	Noninvasive Vagus	Cluster Headache n = 48	Multicenter double-blinded RCT	Confirmation study in Europe, nVNS superior to sham for episodic cluster headache, no difference for total population
Silberstein et al. EVENT (2016) [67]	Noninvasive Vagus	Chronic Migraine n = 59	Multicenter double-blinded RCT	No significant difference in number headache days at 2 mos. Statistically significant decrease from baseline (−7.9%) was seen after 8 months
Tassorelli et al. PRESTO (2018) [74]	Noninvasive Vagus	Episodic Migraine n = 248	Multicenter double-blinded RCT	nVNS superior to sham for freedom from pain 30 and 60 min after attack, repeat tested showed superiority at 120 min

nVNS = noninvasive vagus nerve stimulation.

### 5.5. Peripheral Nerve Field Stimulation

Peripheral nerve field stimulation involves the placement of electrodes near the area of the pain without direct contact to a specific peripheral neve [84]. While research in this area is relatively new, several observational studies have been completed to evaluate this treatment’s efficacy. Verrills et al. evaluated 83 patients who had undergone PNFS targeting the occipital, supraorbital, and infraorbital nerves for chronic daily headache, chronic migraines, and occipital neuralgia [20]. Overall, they found a mean NRS decrease of 4.8, with 68% of the patients experiencing >50% reduction in pain. Furthermore, 23/35 of the patients at follow-up reported a moderate-to-extreme decrease in analgesic use at follow-up, highlighting its possibility for headache treatment.

In 2018, Ishiyama et al. applied C2 PNFS, using electroacupuncture for primary headache [85]. In this observational study of 54 patients, significant decreases in NRS pain was found with C2 PNFS use, alongside decreases in HIT-6 and SDS (self-rating depressing scale), and decreases in monthly headache days. While more research needs to be done for PNFS, this remains a safe and exciting field for further study.

### 5.6. Remote Electrical Neuromodulation

Remote electrical neuromodulation (REN) is a novel technique for the treatment of acute migraine. The device is placed on the upper arm and stimulates peripheral nerves in the region to induce conditioned pain modulation. Yarnitsky et al. published a randomized, double-blinded, sham-controlled multicenter study across 12 sites with 252 adults to evaluate pain relief at 2 h post-stimulation [86]. They found that active stimulation was more effective in pain relief at 2 h versus the sham, sustained up to 48 h after treatment. While further research remains, this suggests REN can be an effective treatment for acute migraines.

## 6. Conclusions

Peripheral nerve stimulation offers promising treatments for intractable headaches. Their success relies on accurate diagnosis and appropriate patient selection. While high-quality evidence does exist regarding its use, additional research is needed in the field. For the physician wishing to utilize PNS in practice, the strongest up-to-date evidence for its use is ONS for treatment of chronic migraines, transcutaneous supraorbital nerve stimulation for migraines, and nVNS for cluster headaches. Preliminary evidence also suggests that interventional therapies may be beneficial as adjuncts to standard-of-care therapies (oxygen, triptans, etc.), thus forming a potential area of study. Looking towards the future, much work remains to bring PNS into the mainstream for headache intervention.

## Figures and Tables

**Table 1:** Classification of headaches.

Primary Headaches	Secondary Headaches
Migraine	Medication overuse headache
Tension-type headaches	Trauma
Trigeminal autonomic cephalalgias	Neurological disorders
○ Cluster headache	Vascular disorders
○ Paroxysmal hemicranias	Central nervous system malignancies
○ SUNCT * and SUNA **	Underlying systemic causes (hypertension, fever, and sinusitis)
○ Hemicrania continua	Posttraumatic headache

* SUNCT: short-lasting unilateral neuralgiform headache attacks with conjunctival injection and tearing. ** SUNA: short-lasting unilateral neuralgiform headache attacks with cranial autonomic symptoms.

**Table 2:** Common PNS complications [15,16,17,18,19,20].

Type	Incidence
Infection	3.6–17.9%
Erosion	4.5–50%
Migration	9–25%
Mechanical Failure	3.6%
Lack of Efficacy	21%

**Table 3:** Nerve targets for neuromodulation and evidence.

Treatment	Quality of Evidence	High Yield Summary
Occipital Nerve Stimulation (ONS)	High quality evidence for chronic migraines (CM) Low quality evidence for occipital neuralgia (ON) Low quality evidence for cluster headaches (CH)	6/7 RCT, 8/8 observational studies with benefit for CM [8,17,21,22,23,24,25,26,27,28,29,30,31,32,33] 7/7 observational studies with benefit for ON [27,34,35,36,37,38,39] 9/9 observational studies with benefit for CH [2,22,40,41,42,43,44,45,46,47]
Supraorbital Nerve Stimulation (SON)	Low quality evidence for trigeminal autonomic cephalgia (TAC), trigeminal neuropathic pain, or craniofacial pain	6/6 observational studies with benefit (few subjects overall) [48,49,50,51,52,53]
Infraorbital Stimulation	Low quality evidence for trigeminal neuropathic pain, craniofacial pain	3/3 observational studies with benefit (few subjects overall) [49,51,52]
Transcutaneous Supraorbital Nerve Stimulation	High quality evidence for treatment of episodic and acute migraines	2/2 RCT, 1/1 observational study with benefit [51,54,55,56]
Transcutaneous Supraorbital and Supratrochlear	Moderate quality evidence for treatment of chronic migraines	1 RCT with benefit for CM [57]
SON and Occipital Stimulation	Low quality evidence for treatment of chronic migraine and hemiplegic migraine	3/3 observational studies with benefit [58,59,60]
Invasive Vagus Nerve Stimulation (VNS)	Low quality evidence for treatment of chronic migraine, cluster headache, chronic headache	4/4 observational studies with benefit (few subjects overall) [61,62,63,64]
Noninvasive Vagus Nerve (nVNS)	Moderate quality evidence for treatment of chronic migraines High quality evidence for treatment of cluster headaches Moderate quality evidence for episodic migraines	2/2 RCT, 2/2 observational with some benefit for CM [65,66,67,68] 3/3 RCT, 2/2 observational with benefit for CH (more evidence for episodic CH) [69,70,71,72,73] 1 RCT with benefit for episodic migraines [74]

**Table 4:** Summary of studies reviewed regarding occipital nerve stimulation.

Author	Study	Design	Results
Weiner and Reed (1999) [39]	Occipital Neuralgia n = 13	Observational	12/17 with >50% pain relief, follow-up 1.5–6 years
Oh et al. (2004) [27]	N = 20 (10 for ON, 10 for migraine)	Observational	>50% pain relief achieved in 100% at 1 month, 94% at 6 months
Kapural et al. (2005) [35]	Occipital Neuralgia n = 6	Observational	Significant decrease in VAS and PDI seen at 3 months
Slavin et al. (2006) [38]	Occipital Neuralgia n = 14	Observational	10/14 with successful PNS trial (>50% pain relief), 7/10 with improved pain control and decrease pain medication intake at follow-up
Johnstone et al. (2006) [34]	Occipital Neuralgia n = 7	Observational	5/7 (71%) with >50% VAS reduction, reduced opioid doses in all patients
Melvin et al. (2007) [36]	Occipital Neuralgia n = 11	Prospective pilot study	91% with reduced medication use, 73% with good to excellent relief
Salmasi et al. (2020) [37]	Occipital Neuralgia n = 3	Observational	Average pain reduction of 50% after 8 months
Vadivelu et al. (2012) [77]	Occipital headaches with Chari I n = 15	Observational	87% with continued pain relief at follow-up (avg 19 months), all with >50% VAS reduction
Popeney et al. (2003) [28]	Chronic migraine n = 25	Observational	Average 89% improvement in MIDAS score, all patients reported headaches well controlled
Matharu et al. (2003) [25]	Chronic migraine n = 8	Observational	100% had good to great pain relief
Lipton et al. PRISM (2009) [23]	Chronic migraine n = 140	Multicenter, double-blinded RCT	Abstract only, no statistically significant difference
Saper et al. ONSTIM (2011) [30]	Chronic migraine n = 67	Multicenter, single-blinded RCT	51 implanted devices, 39% response rate had >50% VAS improvement
Dodick et al. (2014) [8]	Chronic migraine n = 157	Multicenter, double-blinded RCT	52 week results showing significantly reduction of headache days by 6.7, excellent or good headache relief in 65%, significantly decreased MIDAS + Zung pain/distress scores
Serra and Marchioretto (2012) [32]	Chronic migraine n = 30	Single center crossover RCT	Significant improvement in headache intensity/frequency, MIDAS, quality of life for all. Decreased drug use
Slotty et al. (2014) [33]	Chronic migraine n = 8	Single center crossover RCT	Improved VAS, no change in SF-36
Miller et al. (2016) [26]	Chronic migraine n = 53	Observational	45% with >30% reduction in moderate-severe headache days
Mekhail et al. (2017) [17]	Chronic migraine n = 20	Single center double-blinded RCT	60% of patients with >30% reduction in pain, 35% with >50% reduction
Schoenen et al. (2016) [31]	Chronic migraine n = 23	Observational	>30% response in 42% for transcutaneous ONS. Significantly decreased total headache days and migraine days
Liu et al. (2017) [24]	Chronic migraine n = 110	Single center single-blinded RCT	>50% response in group treated with both transcutaneous ONS and topiramate. Significant reduction in headache intensity in all groups vs. sham
Rodrigo et al. (2017) [29]	Chronic migraine n = 37	Observational	Substantial pain reduction in most patients, average VAS decrease of 4.9
Garcia-Ortega et al. (2019) [22]	N = 17 (12 with migraine, 5 cluster)	Observational	Burst ONS with mean reduction 10.2 headache days per month in CM, significant mean reduction in frequency and intensity for cluster
Ashkan et al. (2020) [21]	Chronic migraine n = 112	Observational	Decrease in MIDAS, HIT-6 at follow-up, decreased headache days. 46.7% were satisfied/very satisfied at 24 months
Schwedt et al. (2007) [46]	N = 15 (8 for CM, 3 for cluster, 2 for hemicrania, 2 for post-traumatic HA)	Observational	Improvement in frequency, severity, MIDAS
Trentman et al. (2008) [47]	Cluster Headache n = 10	Observational	50% of patients with >50% reduction in headache frequency or severity
Burns et al. (2009) [40]	Cluster Headache n = 14	Observational	71% with improvement in symptoms
Magis et al. (2011) [44]	Cluster Headache n = 15	Observational	80% with <90% improvement in symptoms
Fontaine et al. (2011) [42]	Cluster Headache n = 13	Observational	77% with >50% improvement in symptoms
Mueller et al. (2011) [45]	Cluster Headache n = 10	Observational	Frequency, duration, severity of attacks reduced in 90% of patient. 100% with improvement in quality of life
Leone et al. (2017) [43]	Cluster Headache n = 35	Observational	66.7% of patients with >50% reduction in frequency at mean 6.1 years follow-up
Fontaine et al. (2017) [41]	Cluster Headache n = 44	Observational	59% with >50% improvement in attack frequency. 70% responsive, 47.8% excellent responders

VAS = pain visual analog scale, PDI = pain disability index, MIDAS = migraine disability assessment score, Zung = pain and distress score (PAD), SF-36 short form health surgery (measure of quality of life).

## References

[1] Stovner L., Hagen K., Jensen R., Katsarava Z., Lipton R., Scher A., Steiner T., Zwart J.-A. (2007). The Global Burden of Headache: A Documentation of Headache Prevalence and Disability Worldwide. Cephalalgia.

[2] Burch R., Rizzoli P., Loder E. (2018). The Prevalence and Impact of Migraine and Severe Headache in the United States: Figures and Trends from Government Health Studies. Headache J. Head Face Pain.

[3] Levin M. (2013). The International Classification of Headache Disorders, 3rd Edition (ICHD III)—Changes and Challenges. Headache J. Head Face Pain.

[4] Gupta R., Fisher K., Pyati S. (2019). Chronic Headache: A Review of Interventional Treatment Strategies in Headache Management. Curr. Pain Headache Rep..

[5] Rozental J.M., Benzon H.T., Raja S., Liu S.S., Fishman S.M., Cohen S.P. (2018). Migraine headache and the trigeminal autonomic cephalalgias. Essentials of Pain Medicine.

[6] Ahmed F. (2012). Headache disorders: Differentiating and managing the common subtypes. Br. J. Pain.

[7] Antony A.B., Mazzola A.J., Dhaliwal G.S., Hunter C.W. (2019). Neurostimulation for the Treatment of Chronic Head and Facial Pain: A Literature Review. Pain Physician.

[8] Dodick D.W., Silberstein S.D., Reed K.L., Deer T.R., Slavin K.V., Huh B.K., Sharan A.D., Narouze S., Mogilner A., Trentman T.L. (2014). Safety and efficacy of peripheral nerve stimulation of the occipital nerves for the management of chronic migraine: Long-term results from a randomized, multicenter, double-blinded, controlled study. Cephalalgia.

[9] Deer T.R., Mekhail N., Petersen E., Krames E., Staats P., Pope J., Saweris Y., Lad S.P., Diwan S., Falowski S. (2014). The Appropriate Use of Neurostimulation: Stimulation of the Intracranial and Extracranial Space and Head for Chronic Pain. Neuromodulation Technol. Neural Interface.

[10] Campbell C.M., Jamison R.N., Edwards R.R. (2013). Psychological Screening/Phenotyping as Predictors for Spinal Cord Stimulation. Curr. Pain Headache Rep..

[11] Deer T.R., Leong M.S., Buvanendran A., Gordin V., Kim P.S., Panchal S.J., Yaksh T.L., Wiese A.J., Janicki P.K., Murinson B.B. (2013). Comprehensive Treatment of Chronic Pain by Medical, Interventional, and Integrative Approaches: The American Academy of Pain Medicine Textbook on Patient Management.

[12] Fishbain D.A., Goldberg M., Meagher R.B., Steele R., Rosomoff H. (1986). Male and female chronic pain patients categorized by DSM-III psychiatric diagnostic criteria. Pain.

[13] Petersen E., Slavin K.V. (2014). Peripheral Nerve/Field Stimulation for Chronic Pain. Neurosurg. Clin. North Am..

[14] Deer T.R., Mekhail N., Provenzano D., Pope J., Krames E., Leong M., Levy R.M., Abejon D., Buchser E., Burton A. (2014). The Appropriate Use of Neurostimulation of the Spinal Cord and Peripheral Nervous System for the Treatment of Chronic Pain and Ischemic Diseases: The Neuromodulation Appropriateness Consensus Committee. Neuromodulation Technol. Neural Interface.

[15] Falowski S., Wang D., Sabesan A., Sharan A. (2010). Occipital nerve stimulator systems: Review of complications and surgical techniques. Neuromodulation Technol. Neural Interface.

[16] McRoberts W.P., Wolkowitz R., Meyer D.J., Lipov E., Joshi J., Davis B., Cairns K.D., Barolat G. (2013). Peripheral Nerve Field Stimulation for the Management of Localized Chronic Intractable Back Pain: Results from a Randomized Controlled Study. Neuromodulation Technol. Neural Interface.

[17] Mekhail N.A., Estemalik E., Azer G., Davis K., Tepper S.J. (2016). Safety and Efficacy of Occipital Nerves Stimulation for the Treatment of Chronic Migraines: Randomized, Double-blind, Controlled Single-center Experience. Pain Pr..

[18] Mobbs R., Nair S., Blum P. (2007). Peripheral nerve stimulation for the treatment of chronic pain. J. Clin. Neurosci..

[19] Slavin K.V. (2011). Technical Aspects of Peripheral Nerve Stimulation: Hardware and Complications. Prog. Neurol. Surg..

[20] Verrills P., Rose R., Mitchell B., Vivian D., Barnard A. (2013). Peripheral Nerve Field Stimulation for Chronic Headache: 60 Cases and Long-Term Follow-Up. Neuromodulation Technol. Neural Interface.

[21] Ashkan K., Sokratous G., Göbel H., Mehta V., Gendolla A., Dowson A., Wodehouse T., Heinze A., Gaul C. (2020). Peripheral nerve stimulation registry for intractable migraine headache (RELIEF): A real-life perspective on the utility of occipital nerve stimulation for chronic migraine. Acta Neurochir..

[22] Garcia-Ortega R., Edwards T., Moir L., Aziz T.Z., Green A.L., FitzGerald J.J. (2019). Burst Occipital Nerve Stimulation for Chronic Migraine and Chronic Cluster Headache. Neuromodulation.

[23] Lipton R.B., Goadsby P., Cady R., Aurora S., Grosberg B., Freitag F., Silberstein S., Whiten D., Jaax K. (2009). PRISM study: Occipital nerve stimulation for treatment-refractory migraine [abstract]. Cephalalgia.

[24] Liu Y., Dong Z., Wang R., Ao R., Han X., Tang W., Yu S. (2017). Migraine Prevention Using Different Frequencies of Transcutaneous Occipital Nerve Stimulation: A Randomized Controlled Trial. J. Pain.

[25] Matharu M., Bartsch T., Ward N., Frackowiak R., Weiner R., Goadsby P.J. (2004). Central neuromodulation in chronic migraine patients with suboccipital stimulators: A PET study. Brain.

[26] Miller S., Watkins L., Matharu M. (2016). Long-term outcomes of occipital nerve stimulation for chronic migraine: A cohort of 53 patients. J. Headache Pain.

[27] Oh M.Y., Ortega J., Bellotte J.B., Whiting D.M., Aló K. (2004). Peripheral Nerve Stimulation for the Treatment of Occipital Neuralgia and Transformed Migraine Using a C1-2-3 Subcutaneous Paddle Style Electrode: A Technical Report. Neuromodulation Technol. Neural Interface.

[28] Popeney C.A., Alo K.M. (2003). Peripheral neurostimulation for the treatment of chronic, disabling transformed migraine. Headache J. Head Face Pain.

[29] Rodrigo D., Acin P., Bermejo P. (2017). Occipital Nerve Stimulation for Refractory Chronic Migraine: Results of a Long-Term Prospective Study. Pain Physician.

[30] Saper J.R., Dodick D.W., Silberstein S.D., McCarville S., Sun M., Goadsby P. (2010). Occipital nerve stimulation for the treatment of intractable chronic migraine headache: ONSTIM feasibility study. Cephalalgia.

[31] Schoenen J., D’Ostilio K., Cosseddu A., Nonis R., Sava S., Magis D. (2016). Transcranial Direct Current Stimulation and Transcutaneous Occipital Nerve Stimulation in Chronic Migraine: A Pilot-Comparison of Therapeutic and Electrophysiological Effects (P2.200). Neurology.

[32] Serra G., Marchioretto F. (2012). Occipital nerve stimulation for chronic migraine: A randomized trial. Pain Physician.

[33] Slotty P., Bara G., Kowatz L., Gendolla A., Wille C., Schu S., Vesper J. (2014). Occipital nerve stimulation for chronic migraine: A randomized trial on subthreshold stimulation. Cephalalgia.

[34] Johnstone C.S.H., Sundaraj R. (2006). Occipital Nerve Stimulation for the Treatment of Occipital Neuralgia-Eight Case Studies. Neuromodulation Technol. Neural Interface.

[35] Kapural L., Mekhail N., Hayek S.M., Stanton-Hicks M., Malak O. (2005). Occipital Nerve Electrical Stimulation via the Midline Approach and Subcutaneous Surgical Leads for Treatment of Severe Occipital Neuralgia: A Pilot Study. Anesthesia Analg..

[36] A Melvin E., Jordan F.R., Weiner R.L., Primm D. (2007). Using peripheral stimulation to reduce the pain of C2-mediated occipital headaches: A preliminary report. Pain Physician.

[37] Salmasi V., O Olatoye O., Terkawi A.S., Hah J.M., Ottestad E., Pingree M. (2020). Peripheral Nerve Stimulation for Occipital Neuralgia. Pain Med..

[38] Slavin K.V., Nersesyan H., Wess C. (2006). Peripheral Neurostimulation for Treatment of Intractable Occipital Neuralgia. Neurosurgery.

[39] Weiner R.L., Reed K.L. (1999). Peripheral Neurostimulation for Control of Intractable Occipital Neuralgia. Neuromodulation Technol. Neural Interface.

[40] Burns B., Watkins L., Goadsby P. (2009). Treatment of intractable chronic cluster headache by occipital nerve stimulation in 14 patients. Neurology.

[41] Fontaine D., Blond S., Lucas C., Regis J., Donnet A., Derrey S., Guegan-Massardier E., Jarraya B., Dang-Vu B., Bourdain F. (2017). Occipital nerve stimulation improves the quality of life in medically-intractable chronic cluster headache: Results of an observational prospective study. Cephalalgia.

[42] Fontaine D., Sol J.C., Raoul S., Fabre N., Geraud G., Magne C., Sakarovitch C., Lanteri-Minet M. (2011). Treatment of refractory chronic cluster headache by chronic occipital nerve stimulation. Cephalalgia.

[43] Leone M., Cecchini A.P., Messina G., Franzini A. (2016). Long-term occipital nerve stimulation for drug-resistant chronic cluster headache. Cephalalgia.

[44] Magis D., Gerardy P.-Y., Remacle J.-M., Schoenen J. (2011). Sustained Effectiveness of Occipital Nerve Stimulation in Drug-Resistant Chronic Cluster Headache. Headache J. Head Face Pain.

[45] Mueller O.M., Gaul C., Katsarava Z., Diener H.C., Sure U., Gasser T. (2011). Occipital Nerve Stimulation for the Treatment of Chronic Cluster Headache—Lessons Learned from 18 Months Experience. Central Eur. Neurosurg..

[46] Schwedt T., Dodick D., Hentz J., Trentman T., Zimmerman R. (2007). Occipital Nerve Stimulation for Chronic Headache—Long-Term Safety and Efficacy. Cephalalgia.

[47] Trentman T.L., Zimmerman R.S., Seth N., Hentz J.G., Dodick D.W. (2007). Stimulation Ranges, Usage Ranges, and Paresthesia Mapping During Occipital Nerve Stimulation. Neuromodulation Technol. Neural Interface.

[48] Amin S., Buvanendran A., Park K.-S., Kroin J., Moric M. (2008). Peripheral Nerve Stimulator for the Treatment of Supraorbital Neuralgia: A Retrospective Case Series. Cephalalgia.

[49] Johnson M.D., Burchiel K.J. (2004). Peripheral Stimulation for Treatment of Trigeminal Postherpetic Neuralgia and Trigeminal Posttraumatic Neuropathic Pain: A Pilot Study. Neurosurgery.

[50] Narouze S.N., Kapural L. (2007). Supraorbital Nerve Electric Stimulation for the Treatment of Intractable Chronic Cluster Headache: A Case Report. Headache J. Head Face Pain.

[51] Slavin K.V., Colpan M.E., Munawar N., Wess C., Nersesyan H. (2006). Trigeminal and occipital peripheral nerve stimulation for craniofacial pain: A single-institution experience and review of the literature. Neurosurg. Focus.

[52] Stidd D.A., Wuollet A.L., Bowden K., Price T., Patwardhan A., Barker S., Weinand M.E., Annabi J., Annabi E. (2012). Peripheral nerve stimulation for trigeminal neuropathic pain. Pain Physician.

[53] Vaisman J., Markley H., Ordia J., Deer T. (2012). The Treatment of Medically Intractable Trigeminal Autonomic Cephalalgia with Supraorbital/Supratrochlear Stimulation: A Retrospective Case Series. Neuromodulation Technol. Neural Interface.

[54] E Chou D., Yugrakh M.S., Winegarner D., Rowe V., Kuruvilla D., Schoenen J. (2018). Acute migraine therapy with external trigeminal neurostimulation (ACME): A randomized controlled trial. Cephalalgia.

[55] Jiang L., Yuan D.L., Li M., Liu C., Liu Q., Zhang Y., Tan G. (2018). Combination of flunarizine and transcutaneous supraorbital neurostimulation improves migraine prophylaxis. Acta Neurol. Scand..

[56] Russo A., Tessitore A., Conte F., Marcuccio L., Giordano A., Tedeschi G. (2015). Transcutaneous supraorbital neurostimulation in “de novo” patients with migraine without aura: The first Italian experience. J. Headache Pain.

[57] Schoenen J., Vandersmissen B., Jeangette S., Herroelen L., Vandenheede M., Gérard P., Magis D. (2013). Migraine prevention with a supraorbital transcutaneous stimulator: A randomized controlled trial. Neurology.

[58] Hann S., Sharan A. (2013). Dual occipital and supraorbital nerve stimulation for chronic migraine: A single-center experience, review of literature, and surgical considerations. Neurosurg. Focus.

[59] Reed K., Black S.B., Banta C.J., Will K.R. (2009). Combined occipital and supraorbital neurostimulation for the treatment of chronic migraine headaches: Initial experience. Cephalalgia.

[60] Reed K.L., Will K.R., Conidi F., Bulger R. (2015). Concordant Occipital and Supraorbital Neurostimulation Therapy for Hemiplegic Migraine; Initial Experience; A Case Series. Neuromodulation Technol. Neural Interface.

[61] Hord E., Evans M., Mueed S., Adamolekun B., Naritoku D.K. (2003). The effect of vagus nerve stimulation on migraines. J. Pain.

[62] Lenaerts M., Oommen K., Couch J., Skaggs V. (2008). Can Vagus Nerve Stimulation Help Migraine?. Cephalalgia.

[63] Mauskop A. (2005). Vagus Nerve Stimulation Relieves Chronic Refractory Migraine and Cluster Headaches. Cephalalgia.

[64] Cecchini A.P., Mea E., Tullo V., Curone M., Franzini A., Broggi G., Savino M., Bussone G., Leone M. (2009). Vagus nerve stimulation in drug-resistant daily chronic migraine with depression: Preliminary data. Neurol. Sci..

[65] Barbanti P., Grazzi L., Egeo G., Padovan A.M., Liebler E., Bussone G. (2015). Non-invasive vagus nerve stimulation for acute treatment of high-frequency and chronic migraine: An open-label study. J. Headache Pain.

[66] Kinfe T.M., Pintea B., Muhammad S., Zaremba S., Roeske S., Simon B.J., Vatter H. (2015). Cervical non-invasive vagus nerve stimulation (nVNS) for preventive and acute treatment of episodic and chronic migraine and migraine-associated sleep disturbance: Preliminary findings from a prospective observational cohort study. J. Headache Pain.

[67] Silberstein S.D., Calhoun A.H., Lipton R.B., Grosberg B.M., Cady R.K., Dorlas S., Simmons K.A., Mullin C., Liebler E.J., Goadsby P.J. (2016). Chronic migraine headache prevention with noninvasive vagus nerve stimulation: The EVENT study. Neurology.

[68] Straube A., Ellrich J., Eren O., Blum B., Ruscheweyh R. (2015). Treatment of chronic migraine with transcutaneous stimulation of the auricular branch of the vagal nerve (auricular t-VNS): A randomized, monocentric clinical trial. J. Headache Pain.

[69] Gaul C., Diener H.-C., Silver N., Magis D., Reuter U., Andersson A., Liebler E.J., Straube A., on behalf of the PREVA Study Group (2016). Non-invasive vagus nerve stimulation for PREVention and Acute treatment of chronic cluster headache (PREVA): A randomised controlled study. Cephalalgia.

[70] Goadsby P.J., de Coo I., Silver N., Tyagi A., Ahmed F., Gaul C., Jensen R.H., Diener H.-C., Solbach K., Straube A. (2017). Non-invasive vagus nerve stimulation for the acute treatment of episodic and chronic cluster headache: A randomized, double-blind, sham-controlled ACT2 study. Cephalalgia.

[71] Marin J., Giffin N., Consiglio E., McClure C., Liebler E., Davies B. (2018). Non-invasive vagus nerve stimulation for treatment of cluster headache: Early UK clinical experience. J. Headache Pain.

[72] Nesbitt A.D., Marin J.C.A., Tompkins E., Ruttledge M.H., Goadsby P.J. (2015). Initial use of a novel noninvasive vagus nerve stimulator for cluster headache treatment. Neurology.

[73] Silberstein S.D., Mechtler L.L., Kudrow D.B., Calhoun A.H., McClure C., Saper J.R., Liebler E.J., Engel E.R., Tepper S.J. (2016). Non–Invasive Vagus Nerve Stimulation for the ACute Treatment of Cluster Headache: Findings from the Randomized, Double-Blind, Sham-Controlled ACT1 Study. Headache J. Head Face Pain.

[74] Tassorelli C., Grazzi L., de Tommaso M., Pierangeli G., Martelletti P., Rainero I., Dorlas S. (2018). Noninvasive vagus nerve stimulation as acute therapy for migraine: The randomized PRESTO study. Neurology.

[75] Choi I., Jeon S.R. (2016). Neuralgias of the Head: Occipital Neuralgia. J. Korean Med. Sci..

[76] Kemp W.J., Tubbs R.S., Cohen-Gadol A.A. (2011). The innervation of the scalp: A comprehensive review including anatomy, pathology, and neurosurgical correlates. Surg. Neurol. Int..

[77] Vadivelu S., Bolognese P., Milhorat T.H., Mogilner A.Y. (2012). Occipital Nerve Stimulation for Refractory Headache in the Chiari Malformation Population. Neurosurgery.

[78] Huff T., Daly D.T. (2021). Cranial Nerve 5 (Trigeminal) [Updated 2020 Nov 19]. StatPearls [Internet].

[79] Simopoulos T., Bajwa Z., Lantz G., Lee S., Burstein R. (2010). Implanted Auriculotemporal Nerve Stimulator for the Treatment of Refractory Chronic Migraine. Headache J. Head Face Pain.

[80] Howland R.H. (2014). Vagus Nerve Stimulation. Curr. Behav. Neurosci. Rep..

[81] Deshpande K.K., Wininger K.L. (2011). Feasibility of combined epicranial temporal and occipital neurostimulation: Treatment of a challenging case of headache. Pain Physician.

[82] Mammis A., Gudesblatt M., Mogilner A.Y. (2011). Peripheral Neurostimulation for the Treatment of Refractory Cluster Headache, Long-Term Follow-Up: Case Report. Neuromodulation Technol. Neural Interface.

[83] Goadsby P., Grosberg B., Mauskop A., Cady R., Simmons K. (2014). Effect of noninvasive vagus nerve stimulation on acute migraine: An open-label pilot study. Cephalalgia.

[84] Chakravarthy K., Nava A., Christo P.J., Williams K. (2016). Review of Recent Advances in Peripheral Nerve Stimulation (PNS). Curr. Pain Headache Rep..

[85] Ishiyama S., Shibata Y., Ayuzawa S., Matsushita A., Matsumura A. (2018). Clinical Effect of C2 Peripheral Nerve Field Stimulation Using Electroacupuncture for Primary Headache. Neuromodulation.

[86] Yarnitsky D., Dodick D.W., Grosberg B.M., Burstein R., Ironi A., Harris D., Lin T., Silberstein S.D. (2019). Remote Electrical Neuromodulation (REN) Relieves Acute Migraine: A Randomized, Double-Blind, Placebo-Controlled, Multicenter Trial. Headache J. Head Face Pain.

